# Multi-Modal Deep Hand Sign Language Recognition in Still Images Using Restricted Boltzmann Machine

**DOI:** 10.3390/e20110809

**Published:** 2018-10-23

**Authors:** Razieh Rastgoo, Kourosh Kiani, Sergio Escalera

**Affiliations:** 1 Electrical and Computer Engineering Department, Semnan University, Semnan 3513119111, Iran; 2 Department of Mathematics and Informatics, University of de Barcelona and Computer Vision Center, 08007 Barcelona, Spain

**Keywords:** hand sign language, deep learning, restricted Boltzmann machine (RBM), multi-modal, profoundly deaf, noisy image

## Abstract

In this paper, a deep learning approach, Restricted Boltzmann Machine (RBM), is used to perform automatic hand sign language recognition from visual data. We evaluate how RBM, as a deep generative model, is capable of generating the distribution of the input data for an enhanced recognition of unseen data. Two modalities, RGB and Depth, are considered in the model input in three forms: original image, cropped image, and noisy cropped image. Five crops of the input image are used and the hand of these cropped images are detected using Convolutional Neural Network (CNN). After that, three types of the detected hand images are generated for each modality and input to RBMs. The outputs of the RBMs for two modalities are fused in another RBM in order to recognize the output sign label of the input image. The proposed multi-modal model is trained on all and part of the American alphabet and digits of four publicly available datasets. We also evaluate the robustness of the proposal against noise. Experimental results show that the proposed multi-modal model, using crops and the RBM fusing methodology, achieves state-of-the-art results on Massey University Gesture Dataset 2012, American Sign Language (ASL). and Fingerspelling Dataset from the University of Surrey’s Center for Vision, Speech and Signal Processing, NYU, and ASL Fingerspelling A datasets.

## 1. Introduction

Profoundly deaf people have many problems in communicating with other people in society. Due to impairment in hearing and speaking, profoundly deaf people cannot have normal communication with other people. A special language is fundamental in order for profoundly deaf people to be able to communicate with others [1]. In recent years, some projects and studies have been proposed to create or improve smart systems for this population to recognize and detect the sign language from hand and face gestures in visual data. While each method provides different properties, more research is required to provide a complete and accurate model for sign language recognition. Using deep learning approaches has become common for improving the recognition accuracy of sign language models in recent years. In this work, we use a generative deep model, Restricted Boltzmann Machine (RBM), using two visual modalities, RGB and Depth, for automatic sign language recognition. A Convolutional Neural Network (CNN) model, using Faster Region-based Convolutional Neural Network (Faster-RCNN) [2], is applied for hand detection in the input image. Then, our goal is to test how a generative deep model, able to generate data from modeled data distribution probabilities, in combination with different visual modalities, can improve recognition performance of state-of-the-art alternatives for sign language recognition. The contributions of this paper are summarized as follows:

(a) A generative model, Restricted Boltzmann Machine (RBM), is used for hand sign recognition. We benefit from the generative capabilities of the network and the need for fewer network parameters to achieve better generalization capabilities with fewer input data. Additionally, we show enhanced performance by the fusion of different RBM blocks, each one considering a different visual modality.
(b) To improve the recognition performance against noise and missing data, our model is enriched with additional data in the form of augmentation based on cropped image regions and noisy regions.
(c) We evaluate the robustness of the proposed model against different kinds of noise; as well as the effect of the different model hyper-parameters.
(d) We provide state-of-the-art results on five public sign recognition datasets.

The rest of this paper is organized as follows: Section 2 reviews the related materials and methods as well as the details of the proposed model. Experimental results on four publicly available datasets are presented in Section 3. Finally, Section 4 concludes the work.

## 2. Materials and Methods

### 2.1. Related Work

Sign language recognition has seen a major breakthrough in the field of Computer Vision in recent years [3]. A detailed review of sign language recognition models can be found in [4]. The challenges of developing sign language recognition models range from the image acquisition to the classification process [3]. We present a brief review of some related models of sign language recognition in two categories:

- Deep-based models: In this category, the proposed models use deep learning approaches for accuracy improvement. A profoundly deaf sign language recognition model using the Convolutional Neural Network (CNN) was developed by Garcia and Viesca [5]. Their model classifies correctly some letters of the American alphabet when tested for the first time, and some other letters most of the time. They fine-tuned the GoogLeNet model and trained their model on American Sign Language (ASL) and the Finger Spelling Dataset from the University of Surrey’s Center for Vision, Speech, and Signal Processing and Massey University Gesture Dataset 2012 [5]. Koller et al. used Deep Convolutional Neural Network (DCNN) and Hidden-Markov-Model (HMM) to model mouth shapes to recognize sign language. The classification accuracy of their model outperformed state-of-the-art mouth model recognition systems [6]. An RGB ASL Image Dataset (ASLID) and a deep learning-based model were introduced by Gattupalli et al. to improve the pose estimation of the sign language models. They measured the recognition accuracy of two deep learning-based state-of-the-art methods on the provided dataset [7]. Koller et al. proposed a hybrid model, including CNN and Hidden Markov Model (HMM), to handle the sequence data in sign language recognition. They interpreted the output of their model in a Bayesian fashion [8]. Guo et al. suggested a tree-structured Region Ensemble Network (REN) for 3D hand pose estimation by dividing the last convolution outputs of CNN into some grid regions. They achieved state-of-the-art estimation accuracy on three public datasets [9]. Deng et al. designed a 3D CNN for hand pose estimation from a single depth image. This model directly produces the 3D hand pose and does not need further processing. They achieved state-of-the-art estimation accuracy on two public datasets [10]. A model-based deep learning approach has been suggested by Zhou et al. [11]. They used a 3D CNN with a kinematics-based layer to estimate the hand geometric parameters. The report of experimental results of their model shows that they attained state-of-the-art estimation accuracy on some publicly available datasets. A Deep Neural Network (DNN) has been proposed by the LIRIS team of ChaLearn challenge 2014 for hand gesture recognition from two input modalities, RGB and Depth. They achieved the highest accuracy results of the challenge, using early fusion of joint motion features from two input modalities [12]. Koller et al. presented a new approach to classify the input frames using an embedded CNN within an iterative Expectation Maximum (EM) algorithm. The proposed model has been evaluated on over 3000 manually labelled hand shape images of 60 different classes and led to 62.8 top-1 accuracy on the input data [13]. While their model is applied not only for image input but also for frame sequences of a video, there are many rooms to improve the model performance in the case of time and complexity due to using HMMs and the EM algorithm. Guo et al. [14] proposed a simple tree-structured REN for 3D coordinate regression of depth image input. They partitioned the last convolution outputs of ConvNet into several grid regions and integrated the output of fully connected (FC) regressors from regions into another FC layer.
- Non-deep models: In this category, the proposed model does not use deep learning approaches. Philomena and Jasmin suggested a smart system composed of a group of Flex sensors, machine learning and artificial intelligence concepts to recognize hand gestures and show the suitable form of outputs. Unfortunately, this system has been defined as a research project and the experimental results have not been reported [15]. Narayan Sawant designed and implemented an Indian Sign Language recognition system to recognize the 26-character alphabet by using the HSV color model and Principal Component Analysis (PCA) algorithm. In this work, the experimental results have not been reported [16]. Ullah designed a hand gesture recognition system using the Cartesian Genetic Programming (CGP) technique for American Sign Language (ASL). Unfortunately, the designed system is still restricted and slow. Improving the recognition accuracy and learning ability of the suggested system are necessary [17]. Kalsh and Garewal proposed a real-time system for hand sign recognition using different hand shapes. They used the Canny edge detection algorithm and Gray-level images. They selected only six alphabets of ASL and achieved a recognition accuracy of 100 [18]. An Adaptive Neuro-Fuzzy Inference System (ANFIS) was designed to recognize sign language by Wankhade and Zade. They compared the performance of Neural Network, HMM, and Adaptive Neuro-Fuzzy Inference System (ANFIS) for sign language recognition. Based on their experimental results for 35 samples, ANFIS had a higher accuracy than the other methods [19]. Plawiak et al. [20] designed a system for efficient recognition of hand body language based on specialized glove sensors. Their model used Probabilistic Neural Network, Support Vector Machine, and K-Nearest Neighbor algorithms for gesture recognition. The proposed model has been evaluated on data collected from ten people performing 22 hand body languages. While the experimental results show high recognition performance, gestures with low inter-class variability use are miss-classified.

In this work, we propose a deep-based model using RBM to improve sign language recognition accuracy from two input modalities, RGB and Depth. Using three forms of the input images, original, cropped, and noisy cropped, the hands of these images are detected using CNN. While each of these forms for each modality is passed to an RBM, the output of these RBMs are fused in another RBM to recognize the output hand sign language label. Furthermore, we evaluate the noise robustness of the model by generating different test cases, including different types of noise applied to input images. Based on the labels of the input images, some states, including all or parts of the output class labels, are generated. Some of the letters, such as Z and Y, are hardly detected because of the complexities in their signs. In this regard, we generate different states in order to have the freedom to ignore these hardly detected letters in some of the states. We expect that the states that do not include the hardly detected letters or digits have good recognition accuracy. The proposed model is trained on the Massey, ASL dataset at Surrey, NYU, and ASL Fingerspelling A dataset and achieves state-of-the-art results.

### 2.2. Proposed Model

**Figure 1:**
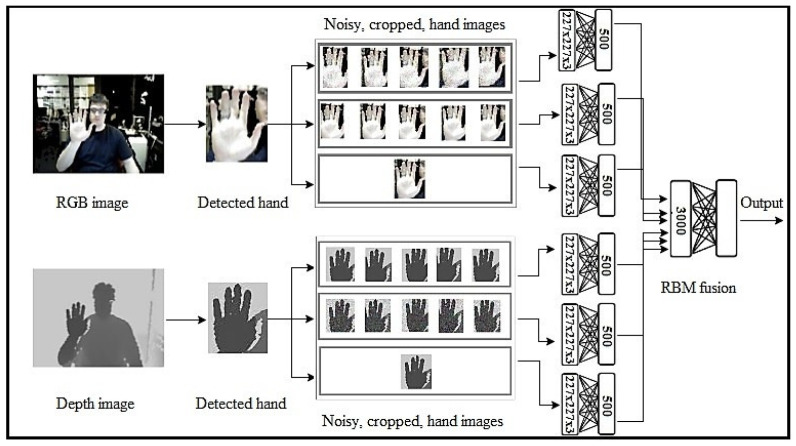
The proposed model.

**Figure 2:**
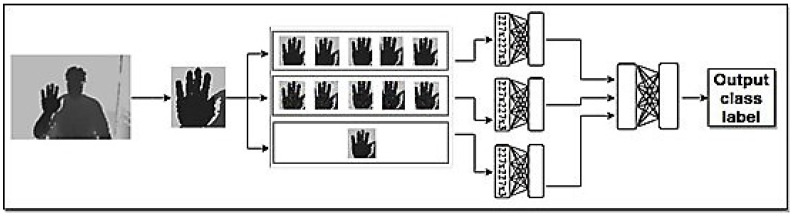
The proposed model in the case of using just depth modality in the input.

**Figure 3:**
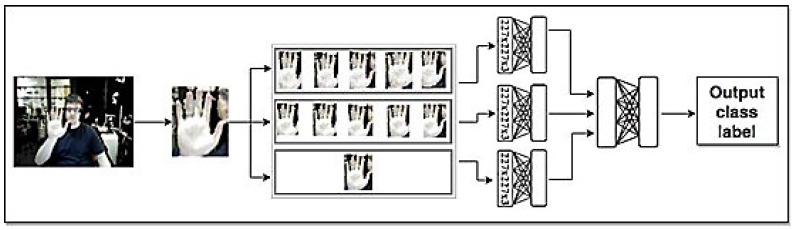
The proposed model in the case of using just RGB modality in the input.

The proposed model includes the following steps:

- Inputs: The original input images are entered into the model in order to extract their features. As Figure 1 shows, we use two modalities, RGB and Depth, in the input images. In the case of one modality in the input images, we use the model illustrated in Figure 2 and Figure 3 for depth and RGB input images.
- Hand detection: To improve the recognition accuracy of the proposed model, a fine-tuned CNN model, based on the Faster-RCNN model [2], is used to detect hands in the input images.
- Crop the images: The input images are cropped from five regions of the image using a CNN.
- Add noise: To increase the robustness of the proposed model, two types of noise, Gaussian and Salt-and-Pepper, are added to input images of the model.
- Enter into the RBM: In the proposed model, we use not only two modalities, RGB and Depth, but also three forms of input image: an original input image, a five cropped input image, and a five noisy cropped input image. For each model, we use these three forms of input image and send them to the RBM. Six RBMs are used in this step as follows:
  First RBM: The inputs of the first RBM are five RGB noisy cropped images. Each of these five noisy crops is separately input to the RBM.
  Second RBM: Five crops of RGB input image are the inputs of the second RBM.
  Third RBM: Only the original detected hand of the RGB input image is considered as the input of third RBM.
  Fourth RBM: Five depth noisy cropped images are separately sent to the fourth RBM.
  Fifth RBM: The inputs of the fifth RBM are five depth cropped images.
  Sixth RBM: The original depth detected hand is considered as the input of the sixth RBM.
- RBM outputs fusion: We use another RBM for fusing the outputs of six RBMs used in the previous step. The outputs of six RBMs are fused and input into the seventh RBM in order not only to decrease the dimension but also to generate the distribution of data to recognize the final hand sign label. In Figure 1, we show how to use these RBMs in our model.

Details of the mentioned parts of the proposed method are explained in the following sub-sections.

#### 2.2.1. Input Image

**Figure 4:**
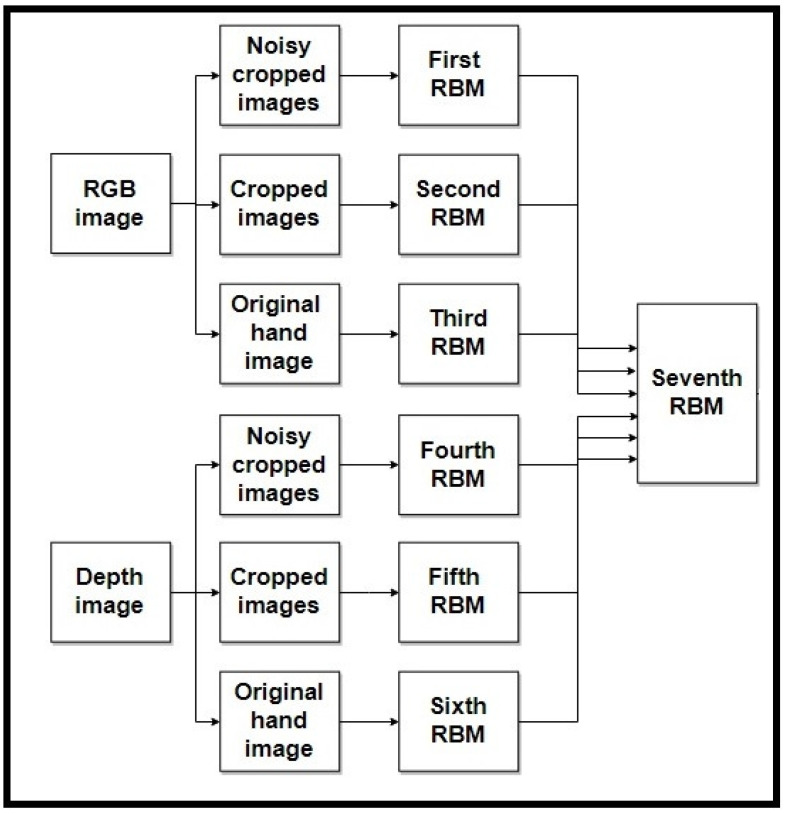
Flowchart of the proposed model.

We use two modalities, RGB and depth, in the input images. In the case that we have only one modality in the input images, we use a part of the model for that input modality. In the proposed multi-modal model, Figure 4, the top part of the model, as seen in Figure 2, is the model for depth inputs and the bottom part, as see in Figure 3, is the model for RGB inputs.

#### 2.2.2. Hand Detecting

The hands in the input image are detected using the fine-tuned Faster-RCNN [2]. Faster-RCNN is a fast framework for object detection using CNN. Faster-RCNN network takes an input image and a set of object proposals. The outputs of this network are the real-valued number-encoded refined bounding-box positions for each of the output classes in the network. Faster-RCNN uses a Region Proposal Network (RPN) to share full-image convolutional features with the detection network, which leads to providing approximately cost-free region proposals. RPN is a fully convolutional network that is used to predict the object bounds. Faster-RCNN achieved state-of-the-art object detection accuracy on some public datasets. In addition, Faster-RCNN has a high frame rate detection on very deep networks such as VGG-16. Sharing the convolutional features has led to decreasing the parameters as well as increasing the detection speed in the network. Due to a high speed and low cost in the object detection, we used the Faster-RCNN to detect the hands in the input images.

#### 2.2.3. Image Cropping

**Figure 5:**
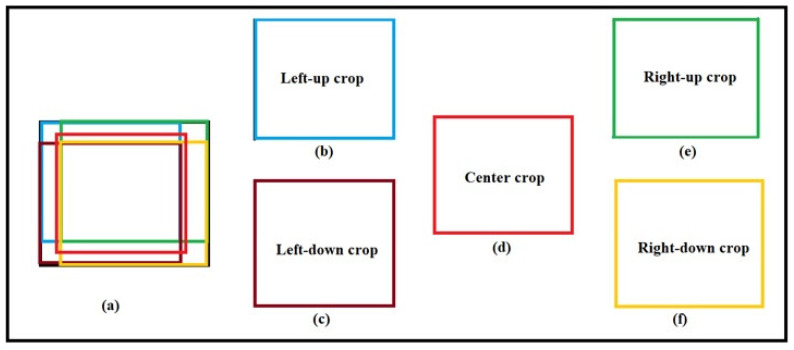
Generating different crops of the input image.

**Figure 6:**
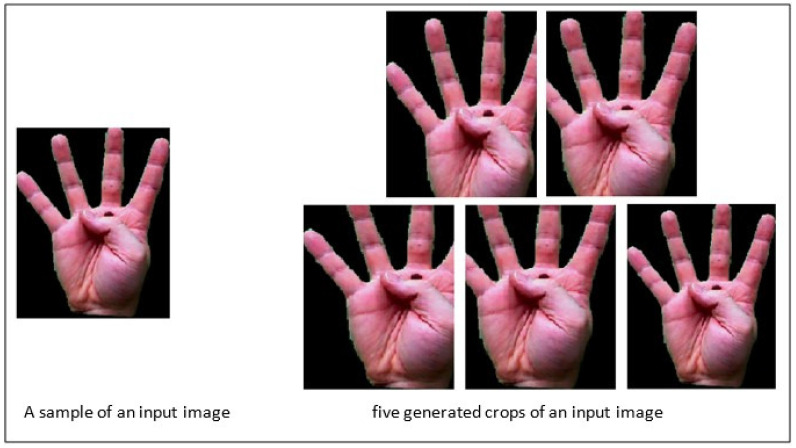
A sample image and generated crops.

To increase the accuracy of the proposed method in recognizing the hand sign language under different situations, different crops of input images are used, as Figure 5 shows. Using different crops is helpful for increasing the accuracy of the model in recognizing input images in situations where some parts of the images do not exist or have been destroyed. In addition, by using these crops, the size of the dataset is increased, being beneficial for deep learning approaches. The proposed method is evaluated by using different numbers of crops to select the suitable number of crops. Furthermore, the proposed method is trained not only on the input images without any crops but also on the cropped images. A sample generating different crops of an image is shown in Figure 6.

#### 2.2.4. Add Noise

**Figure 7:**
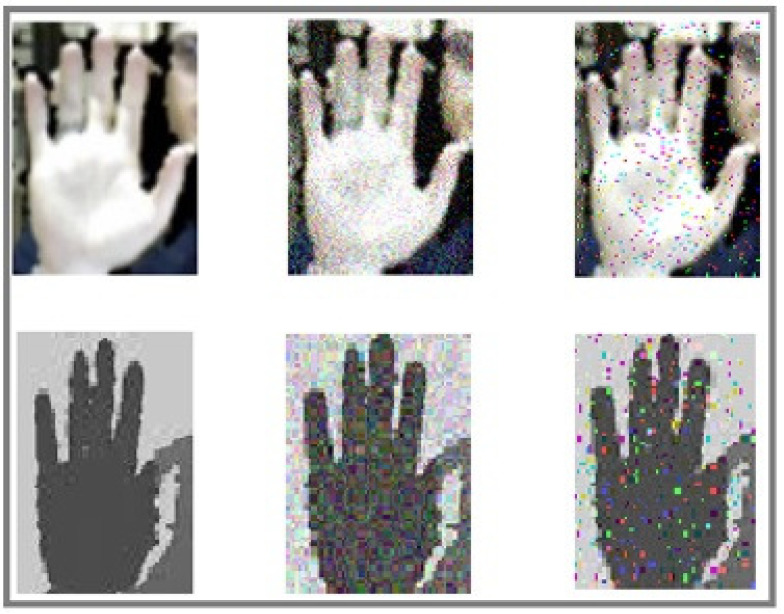
A sample image applying different kinds of noise. (**Left column**): original images, (**Internal column**): Gaussian noise, (**Right column**): Salt-and-pepper noise.

To increase the noise robustness of the proposed method, three types of noise are added to the input images. Figure 7 shows a sample image as well as the applied noises. Gaussian, Gaussian Blur, and Salt-and-Pepper noises are selected due to some beneficial features such as being additive, independent at each pixel, and independent of signal intensity. Four test sets are generated to evaluate the noise robustness of the proposed method as follows:

1. TSet1: In this test set, Gaussian noise is added to the data.
2. TSet2: In this test set, Salt-and-Pepper noise is added to the data.
3. TSet3: In this test set, Gaussian noise is added to one part of data and Salt-and-Pepper noise is added to another part of data.
4. TSet4: In this test set, Gaussian Blur noise is added to the data.

#### 2.2.5. Entry into the RBM

**Figure 8:**
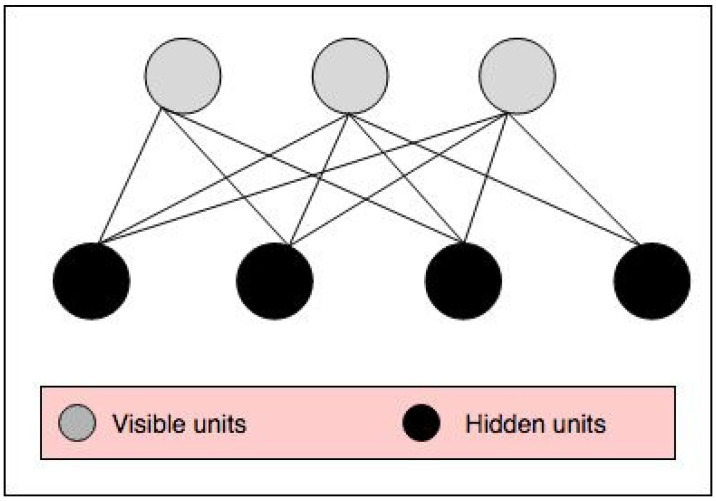
RBM network graph.

RBM is an energy-based model that is shown via an undirected graph, as illustrated in Figure 8. RBM is used as a generative model in different types of data and applications to approximate data distribution. The RBM graph contains two layers, namely visible and hidden units. While the units of each layer are independent of each other, they are conditioned on the units of the other layer. RBM can be trained by using the Contrastive Divergence (CD) learning algorithm. To acquire a suitable estimator of the log-likelihood gradient in RBM, Gibbs sampling is used. Suitable adjustment of the parameters of RBM, such as the learning rate, the momentum, the initial values of the weights, and the number of hidden units, plays a very important role in the convergence of the model [21,22].

**Figure 9:**
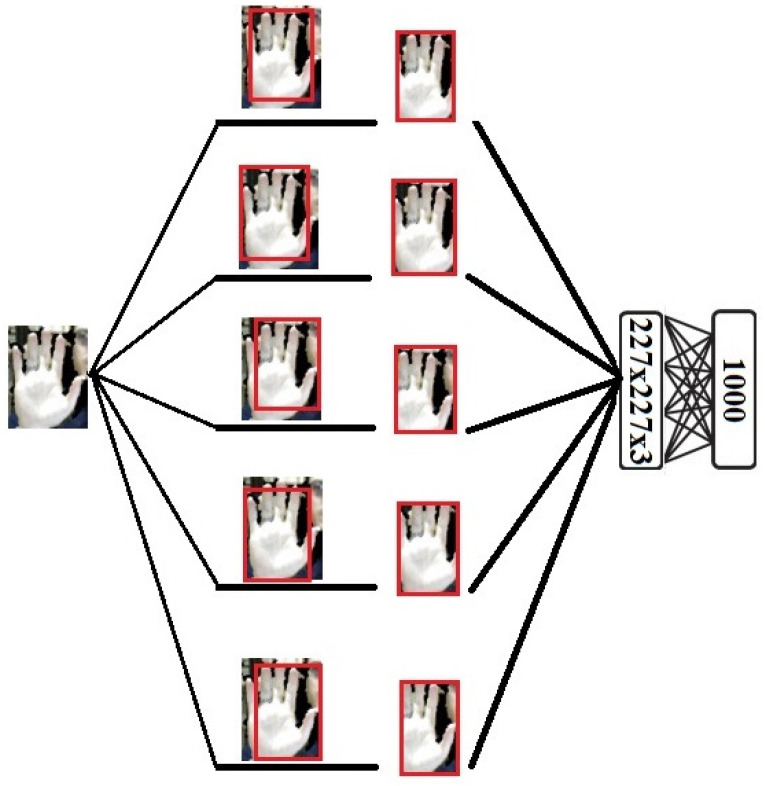
The RGB cropped detected hand inputs of one of the RBMs used in the proposed model.

We are using a reduced set of data where CNN approaches are not able to generalize well. In this case, RBM, a deep learning model with fewer parameters on the generated dataset, can be a good alternative. In the proposed method, we use RBM for hand sign recognition. The achieved results comparing the proposed method with the CNN models shows the outperforming of the RBM model for hand sign recognition on the tested datasets. We use some RBMs in the proposed method for generating the distribution of the input data as well as the recognizing the hand sign label. For each input image modality, we use three RBMs for three forms of input images, which are: original detected hand image, five cropped detected hand images, and five noisy cropped detected hand images. While the input layer of these RBMs includes the size of the 227×227×3 visible neurons, the hidden layer has 500 neurons. Figure 9 shows the RGB cropped detected hand inputs of one of the RBMs used in the proposed model.

#### 2.2.6. Outputs Fusing

**Figure 10:**
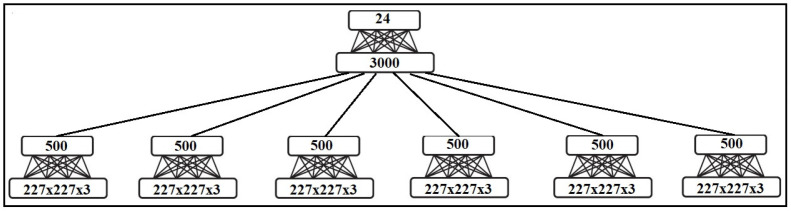
RBM outputs fusing in two-modality inputs of our model.

The outputs of the RBMs, used for each form of the input image for each input modality, are fused in another RBM for hand sign label recognition, while in the case of having just one modality, RGB or depth, we fused three RBM outputs of three input image forms, and fused six RBM outputs in two-modality inputs. Figure 10 shows the RBM outputs fusing for two-modality inputs of our model.

## 3. Results and Discussion

Details of the achieved results of the proposed method on four public datasets are discussed in this section. Results are also compared to state-of-the-art alternatives. Furthermore, we self-compared the proposed model on four used datasets.

### 3.1. Implementation Details

We implemented our model on Intel(R) Xeon(R) CPU E5-2699 (2 processors) with 30 GB RAM on Microsoft Windows 10 operating system and Matlab 2017 software on NVIDIA GPU. Training and test sets are set as defined in the public dataset description for all methods. Five crops of input images are generated and used. We use Stochastic Gradient Descent (SGD) with a mini-batch size of 128. The learning rate starts from 0.005 and is divided by 10 every 1000 epochs. The proposed model is trained for a total of 10,000 epochs. In addition, we use a weight decay of $1\times 10^{-4}$ and a momentum of 0.92. Our model is trained from scratch with random initialization. To evaluate the noise robustness of our model, we use the Gaussian and Gaussian Blur noise with zero mean and variance equal to 0.16. The noise density parameter of the Salt-and-Pepper noise is 0.13. Details of the used parameters in the proposed method are shown in Table 1.

### 3.2. Datasets

**Figure 11:**
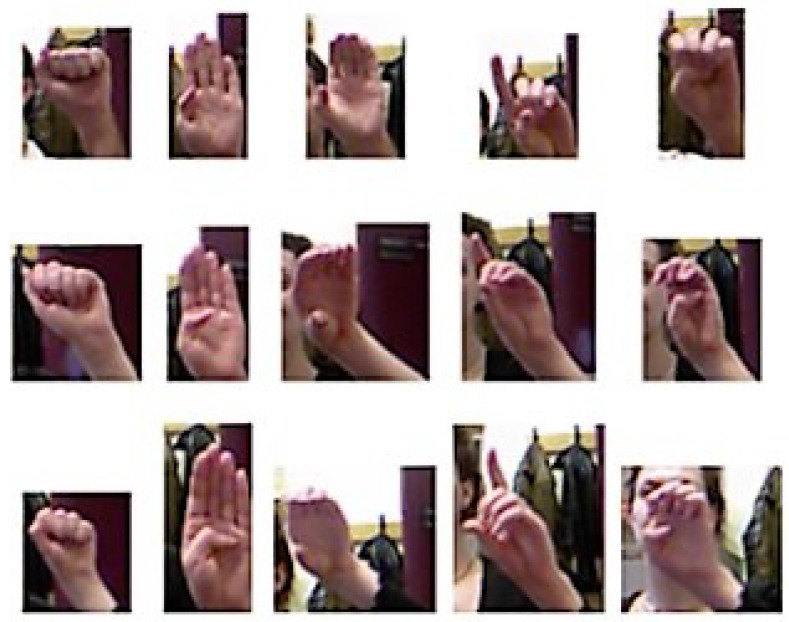
Samples of the American Sign Language (ASL) Fingerspelling A dataset.

The ASL Fingerspelling Dataset from the University of Surrey’s Center for Vision, Speech and Signal Processing [23], Massey University Gesture Dataset 2012 [24], ASL Fingerspelling A [25], and NYU [26] datasets have been used to evaluate the proposed model. Details of these datasets are shown in Table 2. To show the effect of the background in the achieved results, we used not only the datasets without background but also the datasets including background. Figure 11 shows some samples of the ASL Fingerspelling A dataset.

### 3.3. Parameter Evaluation

**Figure 12:**
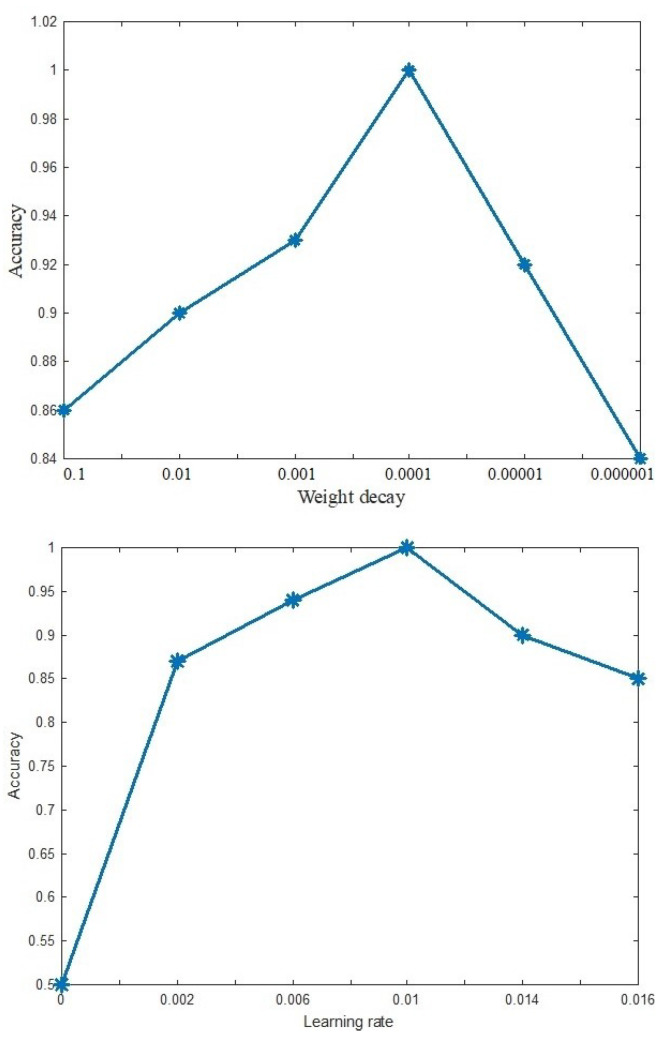
Accuracy versus Weight decay and Learning rate parameters.

Changing some parameters in the proposed method led to different accuracies in the method. Suitable values for the parameters are selected after testing different values for these parameters. Figure 12 shows the effect of changing the learning rate and weight decay parameters in the proposed method. After selecting the best values of the parameters, we fixed and tested the model.

**Figure 13:**
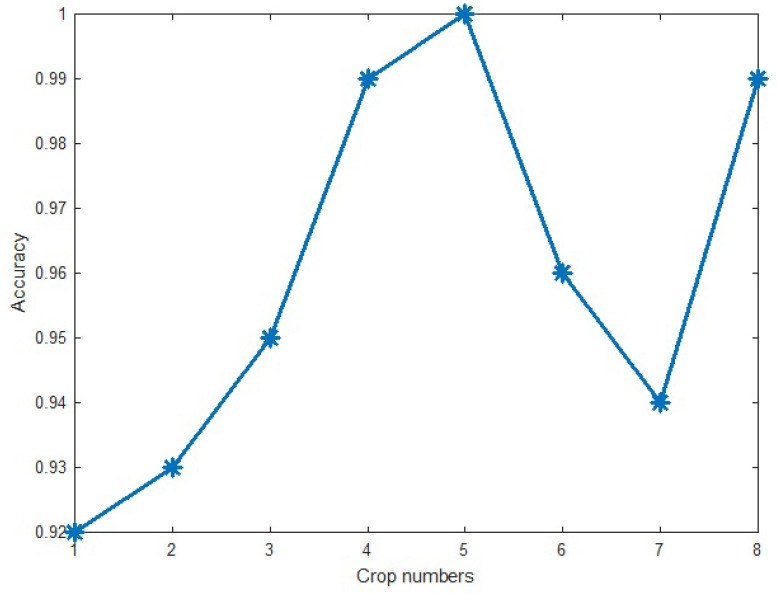
Accuracy versus number of crops of the proposed method on the Massey University Gesture Dataset 2012.

Using the five crops in the training of the proposed method increases not only the size of the dataset but also the robustness of the method in coping with the missed or destroyed parts of the input images. Selecting the suitable number of the crops was done by testing the different values and analyzing the accuracy of the proposed method on the training data. After testing different numbers of crops, the number five was used. Figure 13 shows the best-achieved accuracy of the proposed method in different crops of input images. As Figure 13 shows, while the accuracy of the proposed method monotonically increases in the crop numbers ranging from 1 to 5, the accuracy is approximately fixed in the higher values of the crop number. Due to decreasing of time and cost complexity, five crop numbers were selected.

### 3.4. Self-Comparison

The proposed model is trained on four public datasets for hand sign recognition. We use two modalities in the input images, RGB and Depth. We used accuracy for model evaluation and comparison, defined as follows:

(1) Acc=NT/NT+NF,

with NT being the number of the input samples correctly classified and NF the number of input samples miss-classified. Model has a better accuracy on Massey University Gesture Dataset 2012 than the other datasets used for evaluation. This was predictable because this dataset includes only the RGB images without background in the images. The other datasets, ASL Fingerspelling Dataset from the University of Surrey’s Center for Vision, Speech and Signal Processing, NYU, and ASL Fingerspelling A, have background in their images. Table 3 shows the results of this comparison. Comparison of the results of the proposed model shows that the recognition accuracy of the proposed model on Massey University Gesture Dataset 2012, with RGB input images, were higher than the other used datasets.

### 3.5. Evaluating the Robustness to Noise of the Proposed Method

Four test sets, TSet1, TSet2, TSet3, and TSet4, are generated to evaluate the robustness to noise of the proposed method. Table 4 compares the accuracy of the proposed method in four different states, with the details of the generated test sets being as follows:

1. TSet1: In this test set, the Gaussian noise with zero mean and variance equal to 0.16 is added.
2. TSet2: In this test set, the Salt-and-Pepper noise with noise density equal to 0.13 is added.
3. TSet3: In this test set, the Gaussian noise with zero mean, variance equal to 0.16 is added to one part of data, and Salt-and-Pepper noise with noise density equal to 0.13 is added to another part of data.
4. TSet4: In this test set, the Gaussian Blur noise with zero mean and variance equal to 0.16 is added.

As Table 4 shows, the proposed model achieves higher accuracy on Massey University Gesture Dataset 2012 dataset than with the other used datasets. Due to not having background and occlusion as well as high transparency of the RGB images of this dataset, higher accuracy than the other used datasets with complex background and occlusion in the input images is expected.

### 3.6. State-of-the-Art Comparison

The proposed method is compared with state-of-the-art alternatives in hand sign recognition on four publicly available datasets. Comparison is done under the same conditions of training and testing data partitioning as in previous work, for a fair comparison. As one can observe in Table 5, the proposed model achieves the highest performance in all four datasets.

To evaluate the recognition accuracy of the proposed model for hardly detected characters such as *Z* and *Y*, we generate three categories from the Massey University Gesture Dataset 2012 in order to compare the proposed method with the model suggested by Garcia et al. [5]. The first category includes all 26 characters. The second category includes only 11 characters and ignores the *Z* and *Y*. Finally, the third category includes only 11 characters and ignores the *Z* and *Y*. Details of three categories are as follows:

- Category1: In this category, two models are trained on alphabets to include *a*–*y*.
- Category2: In this category, two models are trained on alphabets to include *a*–*k*.
- Category3: In this category, two models are trained on alphabets to include *a*–*e*.

The results of the comparison of Top-1 and Top-5 accuracies are shown in Table 6 and Table 7. The proposed method significantly outperforms the Garcia and Viesca [5] model in recognition accuracy.

## 4. Conclusions

We proposed the use of RBM as a deep generative model for sign language recognition in multi-modal RGB-Depth data. We showed the model to provide a generalization in instances of low amounts of annotated data thanks to the low number of model parameters. We also showed the model to be robust against different kinds of noise present in the data, and benefitting from the fusion of RGB and Depth visual modalities. We achieved state-of-the-art results in five public sign recognition datasets. However, the model shows difficulty recognizing characters with low visual inter-class variability, such as in the case of the high similarity of hand poses for defining *Z* and *Y* characters. For future work, we plan to further reduce the complexity of the whole ensemble of RBMs by defining isolated simple RBM models that can share information in early training stages. Furthermore, we plan to extend model behavior to deal with image sequences and model spatio-temporal information of sign gestures.

## Figures and Tables

**Table 1:** Details of the parameters in the proposed method.

Parameter	Value	Parameter	Value
Theta for Learning	0.005	Crop numbers	5
Weight Decay	$1\times 10^{-4}$	Batch-size	128
Iteration	100, 1000, 5000, 10,000	Size of the input image	227×227×3
Gaussian Noise Parameters	Mean: 0, Variance: 0.16	Salt-and-pepper noise parameter	noise density: 0.13

**Table 2:** Details of four datasets used for the proposed model evaluation.

Dataset	Language	Class Numbers	Samples	Type
Massey	American	36	2524	Image (RGB)
ASL Fingerspelling A	American	24	131,000	Image (RGB, Depth)
NYU	American	36	81,009	Image (RGB, Depth)
ASL Fingerspelling Dataset of the Surrey University	American	24	130,000	Image (RGB, Depth)

**Table 3:** Recognition accuracy of the proposed model on four datasets.

Dataset	Recognition Accuracy
Massey University Gesture Dataset 2012	**99.31**
ASL Fingerspelling Dataset of the Surrey University	97.56
NYU	90.01
ASL Fingerspelling A	98.13

**Table 4:** Accuracy of the proposed method on four test sets.

Accuracy of Proposed Method	TS1	TS2	TS3	TS4
Massey University Gesture Dataset 2012	95.01	94.94	94.86	**95.36**
ASL Fingerspelling Dataset of the Surrey University	91.09	90.74	90.03	91.18
NYU	85.01	83.84	83.00	85.23
ASL Fingerspelling A	93.84	93.33	92.93	94.04

**Table 5:** State-of-the-art comparison.

Reference	Result	Dataset
[9]	87.00	
[10]	74.00	
[14]	84.40	NYU
[27]	79.40	
**Ours**	**90.01**	
[5]	72.00	Massey University
**Ours**	**99.31**		
[25]	87.00	ASL Fingerspelling A
**Ours**	**98.13**		
[9]	69.00	ASL Surrey
**Ours**	**97.56**		

**Table 6:** Comparison of Top-1 accuracy of the proposed method and Garcia [5] model in three considered categories on Massey University Gesture Dataset 2012.

Top-1 Val Accuracy	Proposed Method	Garcia [5]
Alphabets [*a*–*y*]	**98.91**	69.65
Alphabets [*a*–*k*]	**99.03**	74.30
Alphabets [*a*–*e*]	**99.15**	97.82

**Table 7:** Comparison of Top-5 accuracy of the proposed method and Garcia [5] model in three considered categories on Massey University Gesture Dataset 2012.

Top-5 Val Accuracy	Proposed Method	Garcia [5]
Alphabets [*a*–*y*]	**99.31**	90.76
Alphabets [*a*–*k*]	**99.59**	89.70
Alphabets [*a*–*e*]	99.78	**100**

## Abbreviations

The following abbreviations are used in this manuscript:

RBM	Restricted Boltzmann Machine
CNN	Convolutional Neural Network
DCNN	Deep Convolutional Neural Network
RPN	Region Proposal Network
CD	Contrastive Divergence
SGD	Stochastic Gradient Descent
EM	Expectation Maximum
PCA	Principal Component Analysis
